# Bench-Scale and Full-Scale Level Evaluation of the Effect of Parameters on Cleaning Efficacy of the Firefighters’ PPE

**DOI:** 10.3390/textiles3020014

**Published:** 2023-05-04

**Authors:** Arjunsing Girase, Donald Thompson, Robert Bryan Ormond

**Affiliations:** Textile Protection and Comfort Center, Wilson College of Textiles, North Carolina State University, Raleigh, NC 27695, USA

**Keywords:** NFPA 1851, fireground contaminants, cleaning efficacy, laundering, PPE, PAHs, phenols, phthalates

## Abstract

The National Fire Protection Association (NFPA) 1851 document provides guidelines for firefighters on the care and maintenance of their PPE, including decontamination practices. Firefighters are exposed to various toxic chemicals during fire suppression activities, making effective decontamination crucial for their safety. This study evaluated the efficacy of different washing parameters, including temperature, time, and surfactants, on cleaning outer-shell material contaminated with nine targeted compounds from three different functional groups: phenols, polycyclic aromatic hydrocarbons (PAHs), and phthalates. The study was conducted on both bench-scale and full-scale levels, with contaminated swatches washed in a water shaker bath in the bench-scale evaluation and full-sized washer extractors used in the full-scale evaluation. The results showed that bench-scale washing demonstrated similar trends in contaminant removal to full-scale washing. Importantly, the study highlighted the complexity of removing fireground contaminants from the personal protective ensemble (PPE). The findings of this study have practical implications for the firefighting industry as they provide insight into the effectiveness of different washing parameters for PPE decontamination. Future studies could explore the impact of repeated washing on PPE and investigate the potential for developing more efficient decontamination strategies. Ultimately, the study underscores the importance of ongoing efforts to ensure the safety of firefighters, who face significant occupational hazards.

## 1. Introduction

Firefighters play a critical role in protecting lives and property from the devastating effects of fire. However, their occupation also increases the risk of exposure to a wide range of chemicals that can be toxic to their health. Occupational chemical exposure is one of the probable causes of firefighters’ higher incidences of respiratory disease, heart disease, and cancer than the general population [1–4]. Firefighting PPE has been designed to provide thermal protection. During fire suppression activities, firefighters are exposed to various chemicals, including PAHs and phthalates, among many other classes [5]. PAHs are formed due to the incomplete combustion of materials, and phthalates are ubiquitously used as plasticizers [6].

The following research gaps have been identified in the literature: (1) There is very limited research on the effectiveness of laundering practices used by the firefighting community, particularly in relation to removing specific types of contaminants. (2) Variation in the results of existing studies that have evaluated the effectiveness of laundering practices may be due to differences in sample collection techniques, washing parameters, or other factors. (3) A lack of information on how different washing parameters (e.g., water temperature, detergent type, washing machine type) affect the removal of contaminants from firefighting PPE. (4) Limited research on the comparability between bench-scale testing and full-scale laundering practices in terms of their ability to accurately evaluate the cleaning efficacy of firefighting PPE.

Despite the implementation of laundering practices to decontaminate firefighting gear, limited research has been conducted on the effectiveness of these practices, and the available results indicate high variability in the amounts of targeted compounds across samples. Using a brush with soap and water for on-scene decontamination, Fent et al. showed an 85% reduction in total PAH concentration through wipe sampling of the turnout gear before and after the decontamination procedure. In comparison, only a 23% reduction was shown using the dry-brush technique. This indicates that different decontamination techniques can have different levels of effectiveness [7]. Similarly, Calvillo et al. found a 42% increase in PAH concentration on structural firefighting uniforms using water-only decontamination. However, the increase between pre- and post-wash samples could be due to disparate locations used for sample collection, as the forearm and shoulder areas were selected for sample collection. The spatial variability in these locations and the unknown efficiency of the wipes may have contributed to the increase in concentration after decontamination [8]. Similar studies have shown variations in concentration in pre- and post-laundering samples [9,10]. While Mayer et al. found the contamination for PAHs and organophosphate flame retardants decreased, the concentration of polybrominated diphenyl ethers increased after decontamination practice by 99% in firefighters’ clothing after laundering. However, the results were insignificant due to the small sample size of four [9]. There was a 44% increase in the Benzo[a]Pyrene (BaP) concentration from fabric swatches clipped on the outside of the PPE post-laundering. The results were not statistically significant due to the small sample size [11]. Thus, effective PPE laundering is crucial to decreasing occupational chemical exposure.

Spatial variability in the distribution of contaminants on firefighting PPE is a significant challenge for evaluating the effectiveness of laundering practices. It is crucial to ensure that all areas of the gear are thoroughly cleaned to reduce the risk of exposure to toxic chemicals. Still, the effectiveness of decontamination techniques can be compromised by the spatial variability of contaminants. It can be caused by a variety of factors, including the location and type of exposure to the contaminants during firefighting activities, variations in the materials used in the PPE, and the design and fit of the PPE [12]. Traditional decontamination methods, such as washing with soap and water, may not effectively remove contaminants from hard-to-reach areas of the gear. For example, Calvillo et al. (2019) found that water-only decontamination resulted in higher concentrations of PAHs on the uniform after washing, possibly because contaminants were not effectively removed from areas such as the shoulders and forearms [8].

The issue of spatial variability in the distribution of contaminants on firefighting PPE has important implications for the development of effective decontamination protocols. Developing effective decontamination protocols for firefighting PPE is a complex task. It requires consideration of factors such as the type of contaminants present, the effectiveness of various decontamination methods, and the potential for spatial variability in the distribution of contaminants on the gear. In addition, decontamination protocols need to be practical and cost-effective to implement on a large scale. The small sample size and spatial variation due to uneven contamination made the analysis of the washing practices incomplete. A specified number of replicates will help assess the experiments’ variability. It will help in inferring the significance of the effects of the factors involved in the study. Thus, a standard washing procedure is required to assess the specimens. A bench-scale washing procedure can be used as a standard test for evaluating cleaning efficiency. A bench-scale washing test includes washing small samples in a laboratory environment. Bench-scale washing has several advantages, including being economical and efficient, and all the parameters can be controlled. Over the years, bench-scale washing protocols have been implemented to compare surfactants and washing techniques in assessments of the removal of PAHs [13,14]. Thus, implementing bench-scale washing in evaluating current washing procedures for firefighter gear is justified.

To reduce the risks of occupational chemical exposure for firefighters, it is crucial to optimize laundering practices. The current research study is novel in several ways. Firstly, it tests the consistency of the bench-scale level washing approach by using it for consecutive experiments. Secondly, it aims to evaluate the effectiveness of specific washing parameters, such as detergent type, wash cycle duration, and temperature, in removing targeted fireground contaminants from outer shell material used in firefighting PPE in a controlled environment. This research gap is crucial, as there is limited research on how these specific parameters affect the removal of contaminants from firefighting PPE, despite the risks of occupational chemical exposure to firefighters. This study also includes utilizing a bench-scale approach to evaluate the cleaning efficacy of decontamination practices for firefighting PPE. This approach allows for controlled testing of various parameters and is an economical and efficient way to evaluate the effectiveness of laundering practices. Moreover, while bench-scale testing has been utilized in other industries, it is not widely used in evaluating firefighting PPE laundering practices, making this study highly innovative.

Finally, the study assesses the comparability between bench-scale testing and fullscale laundering practices. Comparing the results obtained from both approaches, this study provides more accurate evaluations of the effectiveness of laundering practices. This comparison is crucial to ensuring that the results obtained from bench-scale testing can be extrapolated to full-scale laundering practices, providing a more accurate evaluation of the effectiveness of laundering practices.

Overall, this research study’s innovative approach provides valuable insights into how laundering practices can be optimized to reduce the risks of occupational chemical exposure for firefighters, ultimately improving their health and safety. The study’s novel methodology and focus on specific washing parameters make it a valuable contribution to the field of firefighting PPE laundering practices.

## 2. Materials and Methods

### 2.1. Testing Variation in Bench-Scale Washing Method

In this study, we aimed to test the consistency of the bench-scale washing method. We used two commercial detergents (CD-1 and CD-2) to wash three contaminated fabric samples on three different days for 18 samples. CD-1 is commonly used in the firefighting community, while CD-2 is a regular home laundry detergent.

We contaminated the fabric swatches, made from PBI Max^™^ Gold (7 oz.), with a repeater pipette (Eppendorf, Hamburg, Germany) to dispense 100,000 ng of each contaminant. We spiked ten droplets, each with a volume of 5 μL, onto each swatch. The swatches were then placed into 250-mL Erlenmeyer flasks with 4.3 g of glass beads added to provide mechanical agitation. The targeted contaminants list provided in Table 1 is similar to the one described in Girase et al. (2022) [15]. The octanol-water partition coefficient values (KOW) represent the compound’s solubility in octanol and water. Higher KOW values indicate higher affinity towards octanol, which also signifies hydrophobicity.

To ensure complete immersion of the fabric, we used a high liquor ratio of 142:1, which required 100 mL of water to wash the 0.7 ± 0.03 g fabric swatches. We washed the samples at 300 RPM at 40 °C for 60 min, following which we rinsed the samples with 100 mL of clean water for 10 min at room temperature. We then air-dried the samples for 24 h before extracting them with a pressurized solvent extractor and analyzing them using GC-MS. The sample set of experimental design for testing consistency is provided in Table 2

We calculated the cleaning efficiency using Equation (1), similar to Girase et al. (2022) [15]:

1 $$\text{Cleaning efficiency}(\text{\%})=\frac{\left(\text{Original concentration}(\text{Cc})-\text{post washing concentration}(\text{Cw})\right)}{\text{Original concentration}}*100$$

### 2.2. Experimental Design for Evaluating the Effect of Washing Parameters on the Cleaning Efficiency on the Bench-Scale

The primary objective of this study was to evaluate the impact of washing parameters on the cleaning efficiency of turnout gear materials when performed according to the NFPA 1851 standard. These parameters include the detergents being used by firefighters as well as washing temperature and duration. The NFPA 1851 standard has prescribed some guidelines that contain important constraints: (1) Temperature should not exceed 40 °C; (2) the pH of the detergent should be between 6 and 10.5 and should not contain chlorine bleaching agents or any oxidizing agents; and (3) the G-force for machine cleaning should be less than 100 G. Considering all the constraints, we performed a full factorial experimental design using JMP Pro 15 software (15.2.0, SAS Institute Inc., Cary, NC, USA). The important parameters considered were temperature, washing duration (time), and surfactant, as shown in Table 3. Of these three parameters, time and temperature are continuous variables, while surfactant is considered categorical. The design of experiments (DOE) was full factorial and contained random sampling. Every experiment was performed in triplicate; the total number of experiments was 24. The prediction profiles were used to study individual parameters’ effects on removing contaminants. Similarly, interaction plots were used to study the parameters’ cross-effects on removing contaminants. The goals were: (1) To evaluate the cleaning efficiency of washing according to NFPA 1851. (2) To study the effect of variation in temperature, washing duration, and surfactant choice on cleaning efficiency.

### Temperatures:

40 °C was chosen since that was the upper limit for washing according to the NFPA 1851 standard. A temperature of 65 °C was added to study the effect of higher temperatures on removing contaminants.

### Time:

There is no particular constraint on the washing duration, but different independent service providers (ISPs) have different washing durations. Hence, the 15- and 60-min cycles were added to study the effect of the short and long washing cycles.

### Surfactants:

The commercially available detergents (CD) CD1 and CD2 were chosen since both were used to validate the bench-scale method. Additionally, CD1 is a popular surfactant in the firefighting community, and CD2 is a regular home laundry surfactant. Choosing these would help us understand the effect of different surfactants on removing different contaminants. The different ingredients for the surfactants are provided in Table 4.

### 2.3. Bench-Scale Washing Method

To ensure consistency with the validation experiments described in Section 2.1, the bench-scale washing method followed a similar process. Each swatch was contaminated with 60,000 ng of the targeted contaminants, the amount specified in the NFPA 1851 standard. This level of contamination was chosen at 60,000 ng since it is also the amount present in the NFPA 1851 standard. To save resources, the amount of contaminant on the fabric was 2400 ng/cm^2^, which was in the middle of the calibration curve. The calibration curve’s midpoint would help reduce the deviations that might occur at larger concentrations.

### 2.4. Full-Scale Washing Method

To assess the effectiveness of bench-scale washing compared to the full-scale washer-extractor commonly used in fire departments and ISPs, a primary objective of this research was to design experiments with similar parameters for both methods.

#### 2.4.1. Sample Preparation for Full-Scale Washing Method

Five unused turnout jackets were modified by attaching hook-and-loop swatches on the outer shell material at strategic locations to hold the test fabric swatches. A total of eight patches were stitched, with four on the front torso, one on each sleeve, and two on the back capable of holding test samples. Only five swatch locations were used due to resource limitations, selected randomly to ensure that at least one swatch was located on the sleeve and one on the back of the garment in each experiment. The study focused on evaluating the outer shell material, and the test samples, contaminated with the master mix of fireground contaminants, were cut from a new roll of PBI Max™ Gold (7 oz.) and were 5 cm × 5 cm in size. The level of contamination was maintained at 60,000 ng to ensure consistency with the bench-scale DOE.

#### 2.4.2. Full-Scale Extractor Method

In the full-scale washing method, we used a UNIMAC 45-lb. (Model no. UWT045D4) washer-extractor that was fully programmable for temperature and washing duration, with parameters similar to those described in Table 3. The contamination and washing procedures were identical to those described as the “Conventional washing protocol” by (Girase et al., 2022) [15].

For CD1, the recommended amount was 180 mL for a 45 lb. load, but since the total load, including the ballast material, was 30 lb., the calculated dosage for CD1 was 120 mL. A similar amount was used for CD2, since the MSDS did not provide any recommended amount for CD2.

### 2.5. Chemical Analysis

The chemical analysis included a pressurized solvent extractor (BUCHI^®^ E-916) and gas chromatography mass spectrometry (Agilent 5977). The detailed procedure is given in Girase et al. (2022) [15].

## 3. Results and Discussion

### 3.1. Consistency of the Bench-Scale Washing Procedure

**Figure 1.**
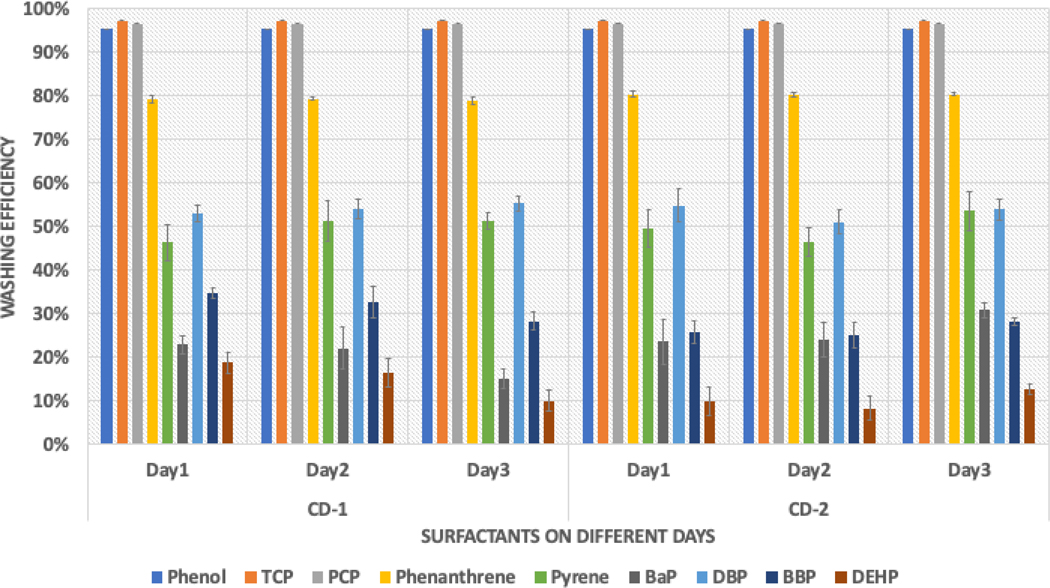
Consistency of the bench-scale washing method.

Figure 1 presents the results of the washing experiment for CD1 and CD2. The graph shows the average washing efficiencies (%) of different compounds for both surfactants over three days. To calculate washing efficiency, the percentage of the initial contamination that was removed after washing was determined. The standard errors are indicated by the error bars in the graph. Since the chromatograms of phenols were not present in the post-washed samples, the washing efficiency of phenols was calculated using LOQ/2 values. The washing efficiencies for both surfactants were consistent across the three days, indicating the reproducibility of the experiment.

**Figure 2.**
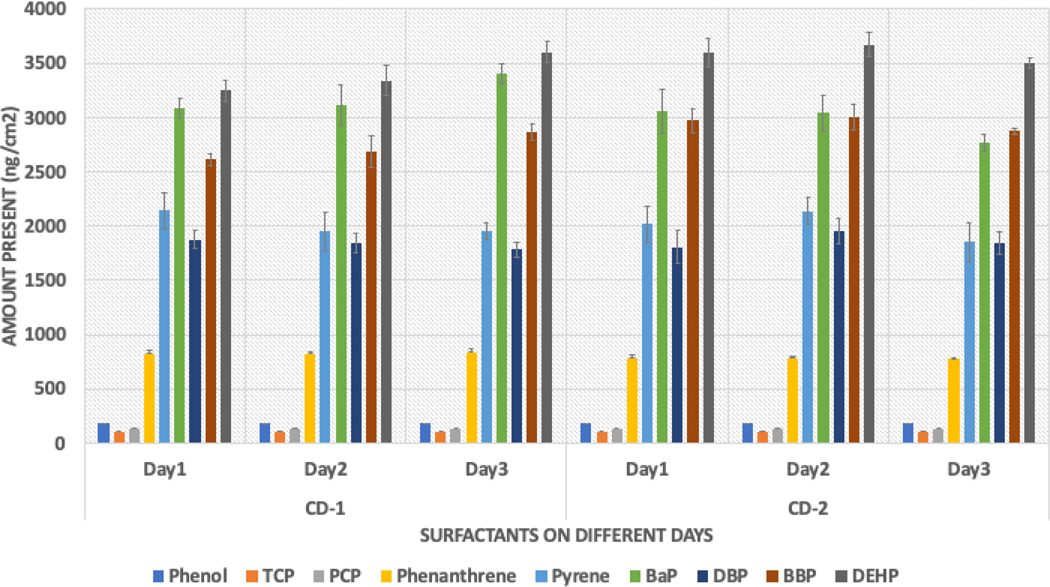
Average contamination present on samples after washing.

The study found an interesting trend in which the average washing efficiency decreased as the polarity of the compounds decreased within any chemical group, as observed from phenanthrene to BaP and DBP to DEHP. The level of contamination spiked on each fabric was 4000 ng/cm^2^, and Figure 2 shows the average contamination present after washing. The study also found that the polarity of the contaminants played a crucial role in cleaning efficiency. Water was used as a cleaning solvent in the washing experiment, and the trend was reversed. Compounds such as DEHP and BaP were present in amounts greater than 3000 ng/cm^2^, consistent with both surfactants. Further investigation is required to examine the effects of various parameters on cleaning efficiency.

### 3.2. Bench-Scale Evaluation of the Various Washing Parameters

**Figure 3.**
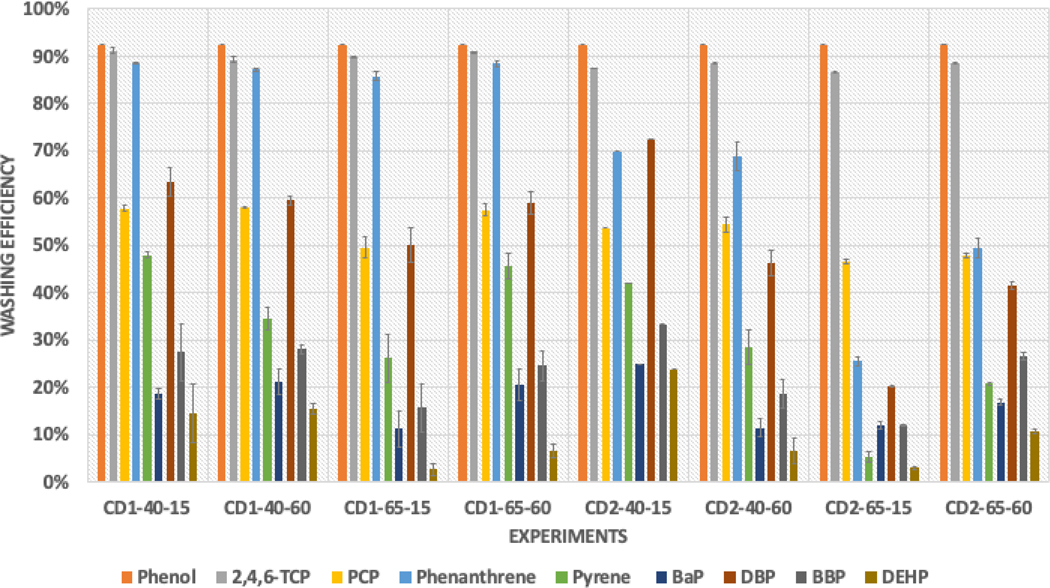
Average washing efficiencies for different combinations of parameters (bench-scale).

The average washing efficiencies of the targeted contaminants are shown in Figure 3. Outliers due to experimental artifacts were observed in the data for two swatches tested with the combination CD2-40-15, and the washing efficiency of phenol compounds was calculated using the LOQ/2 values. Common trends across all combinations indicated that (1) the average washing efficiencies for phenols were higher than those for PAHs and phthalates, and (2) within each chemical group, washing efficiency decreased as the KOW value increased. KOW values measure the relative solubility of a compound in water and octanol, with higher values indicating a greater affinity for octanol, which is a nonpolar solvent. This suggested that the polarity of the cleaning solvent played a significant role in removing contaminants from the fabric. The surfactants less efficiently removed compounds with higher KOW values as they were less soluble in the aqueous cleaning solution. Therefore, consideration of the KOW values of the targeted contaminants should be taken into account when selecting the appropriate cleaning solvent to achieve optimal washing efficiency. This suggested that the polarity of the cleaning solvent played a significant role in removing contaminants from the fabric.

**Figure 4.**
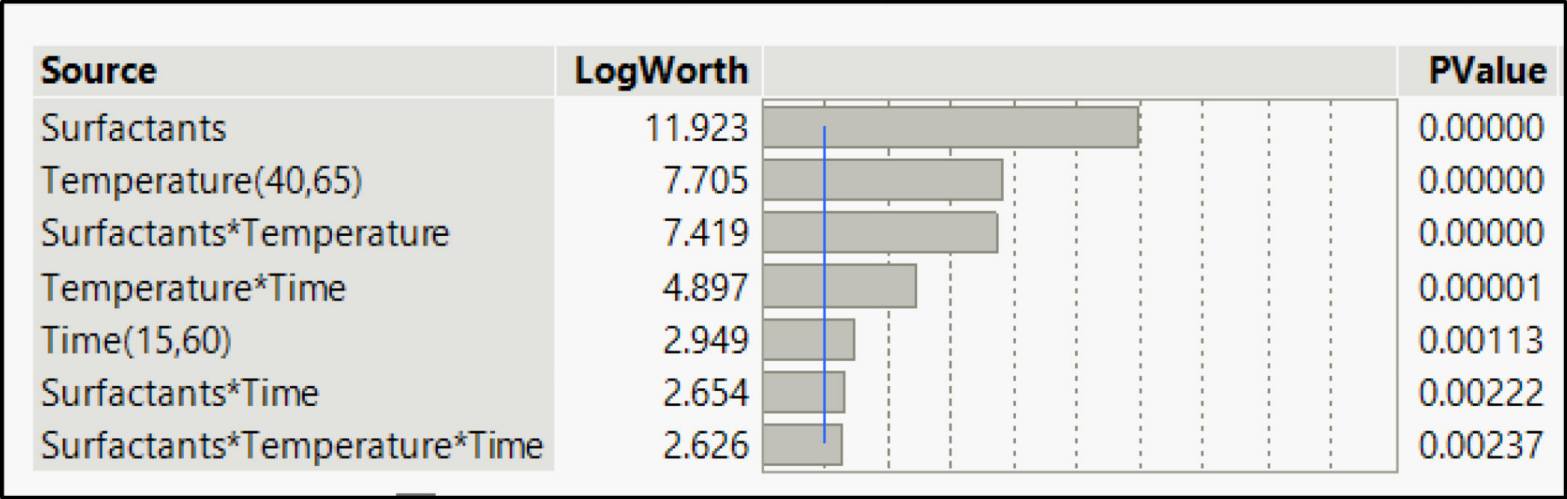
Effect summary for the entire mix.

To study the effects of variation in parameters, the analysis of all the data was performed using JMP pro15. The fit model test was incorporated for individual analysis of the contaminants. The summary of the effects of various parameters is given in the following Figure 4. The LogWorth value is the negative logarithmic value of the *p*-value: −log_10_(*p*-value). A LogWorth value greater than 2 of an effect indicates that the effect has a significant impact at the *p* = 0.01 level, thus indicating the importance of all the parameters on the removal of compounds. The *p*-value is a probability value that describes how likely it is that the data would have occurred randomly. Thus, a high LogWorth value indicated a very low probability of random results being generated or that the effect could have occurred by chance. The summary showed that all the parameters and their cross-effects have a significant impact on removing compounds when they are considered together in the mix. The chemistry of different functional groups and the competitive effects of the chemicals need to be considered for the removal of contaminants from the same mix. The effect summary shown below in Figure 4 is for all the chemicals in the mix. The surfactant displayed a high LogWorth (11.92) value as compared to the other effects. The difference was significant when compared with LogWorth values of other parameters. This high value indicated that the chemistry of contaminants with surfactants played a significant role in the removal of contaminants from the fabric. Thus, the choice of surfactants was a very important parameter in removing the targeted contaminants when considered all together in a mix. The mix contained targeted contaminants from three different classes, thus indicating that the chemical nature of the contaminants needed to be studied while deciding the washing parameters. The effect summary for individual classes of PAHs, phthalates, and phenols is provided in the Supplementary Information (SI) section.

The individual parameters and their effects on different chemical groups are explained in detail below.

#### 3.2.1. Surfactants

**Figure 5.**
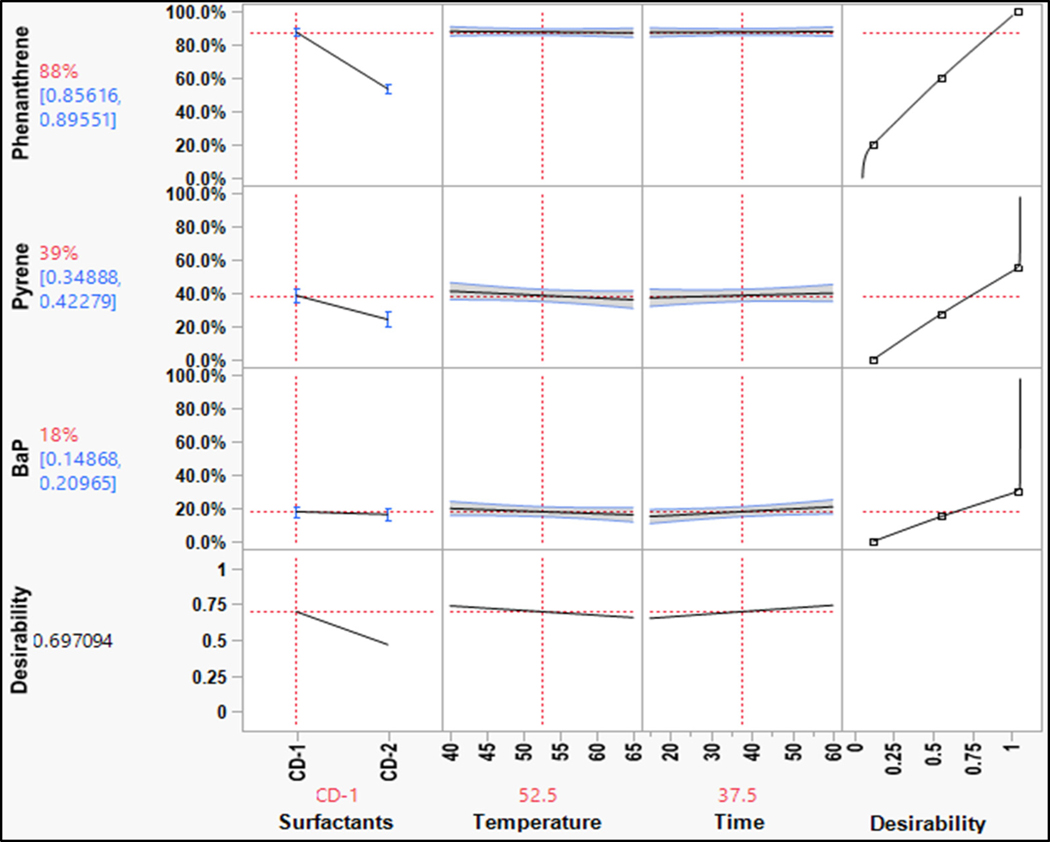
Prediction profile for PAHs (bench-scale).

From the effect summaries, it was evident that the choice of surfactant had a significant impact on removing contaminants, especially PAHs and phenols. The prediction profile of the PAHs for various parameters is shown in Figure 5. The washing efficiency decreases as the number of rings in PAHs increases, which is related to the hydrophobicity of the compound [16]. The responses shown in Figure 5 indicated that overall, CD1 proved to be better than CD2 at removing PAHs. The D-limonene in CD1 is a non-polar compound that effectively removes PAHs since PAHs have a high octanol-water partition coefficient and are non-polar (Table 1). Non-ionic surfactants have been shown to solubilize PAHs effectively [17]. This is consistent with the data since CD1 had a non-ionic surfactant that helped in solubilizing PAHs. For phenanthrene and pyrene, there is a steep decline in washing efficiencies for changes in detergent from CD1 to CD2, indicating that the simple PAHs are more sensitive to changes in surfactant. This highlighted the fact that mixed surfactant systems of anionic and non-ionic surfactants in CD may not be effective in removing PAHs. BaP did not respond positively to any surfactant since it is highly non-polar.

**Figure 6.**
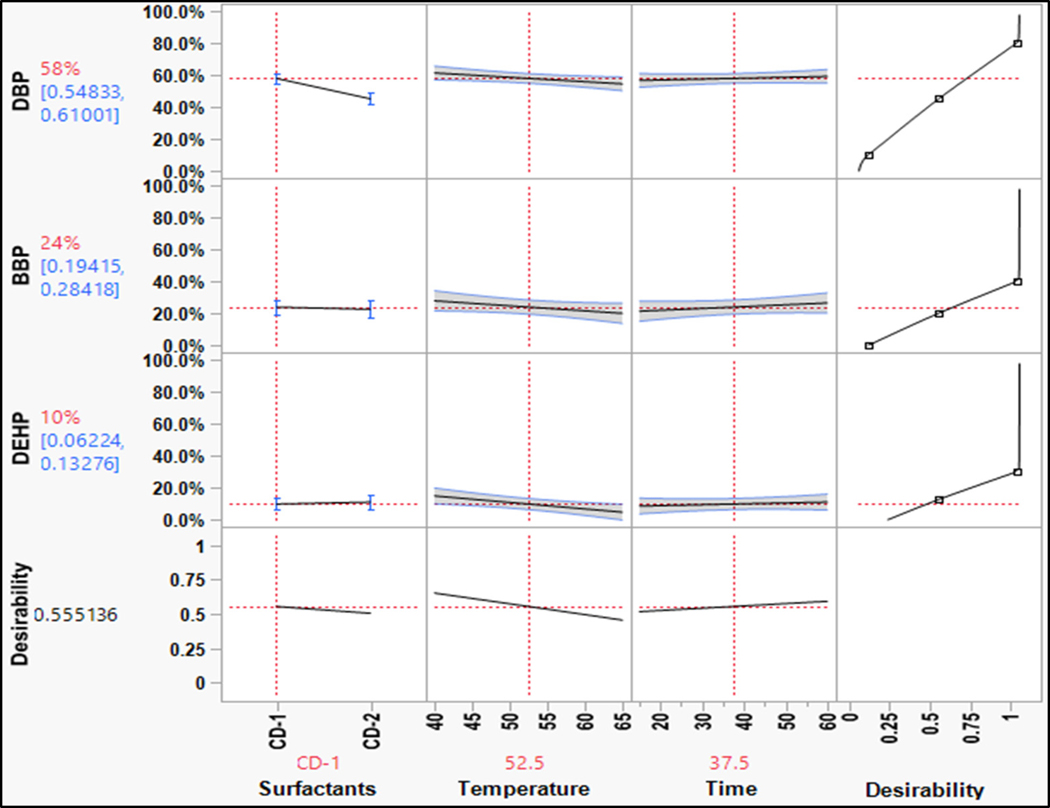
Prediction profile for phthalates (bench-scale).

For phthalates, the overall washing efficiency is low as compared to the other groups (Figure 6). Similar to PAHs, the washing efficiency decreased from simple phthalates to complex phthalates. The KOW value increased as the alkyl chain length increased, indicating an increase in hydrophobicity [18]. The trend in the removal of phthalates was similar to that of PAHs. For a change in surfactant from CD-1 to CD-2, DBP showed a steep declining slope, which indicated that simple phthalates such as DBP were sensitive to changes in surfactants. The D-limonene and non-ionic surfactant removed simpler phthalates effectively, similar to phenanthrene. This indicated that the phthalates would not partition easily in aqueous solutions just by using conventional surfactants. The hydrophobic cores of surfactant micelles can help in desorbing the hydrophobic compounds, but the lower concentration of surfactant limited the availability of these spaces; thus, overall washing efficiency was low [19,20].

**Figure 7.**
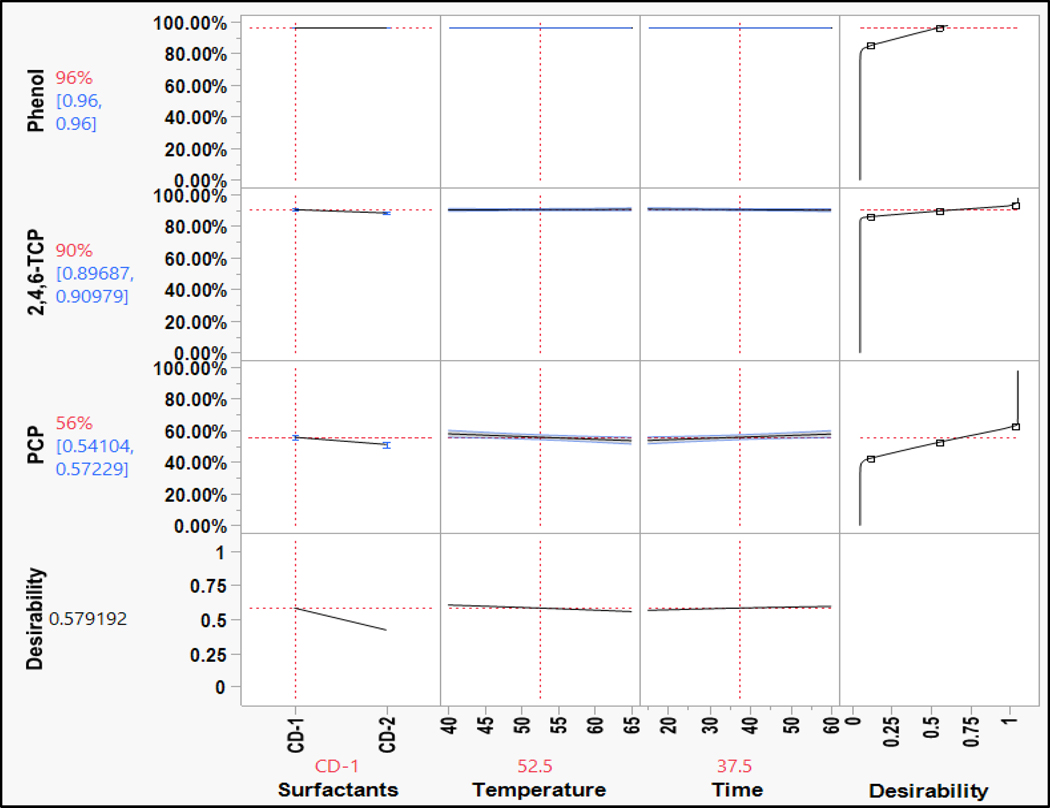
Prediction profile for phenols (bench-scale).

The overall washing efficiency for phenols was high compared to the other chemical groups since phenols are highly polar (Figure 7). The phenol compound is moderately soluble in water. The washing efficiency of phenol was calculated using the LOQ/2 value. Thus, phenol was washed out well beyond the detection limits of the analytical method used. Phenols might form soluble salts with surfactants that help in their removal. The substitutions of compounds on the phenolic ring increase the stability of the ring, which increases the hydrophobicity. Hence, the removal efficiency of the chlorinated phenols decreased in the following order: phenol > 2,4,6-TCP > PCP. The results of phenols for surfactant variation in the parameters were fairly consistent.

#### 3.2.2. Temperature

The temperature was the second important parameter, as seen from the effect summary in Figure 4. The prediction profiles alone did not show any significant change in the trends of washing efficiency with the increase in temperature. For pyrene and BaP, a slight decline is observed with an increase in temperature. The effect summary specifically for PAHs showed that the cross-effect of surfactant and temperature had a comparable LogWorth value (Figure S1). The effect summaries for phthalates and phenols are provided in Figure S2 and Figure S3 respectively. Thus, the interaction plots displayed in Figure S4 highlighted the cross effects of temperature, surfactant, and time. The washing efficiency of the CD2 decreased drastically at higher temperatures. The hydrophilicity of the anionic surfactant increased, while the reverse effect is generally observed in the non-ionic surfactant with increasing temperature [21]. Hence, the washing efficiency decreased drastically for CD2 when compared to CD1 with an increase in temperature. The higher temperatures did not significantly impact the removal of phenanthrene and BaP. This might be because phenanthrene is a simple PAH and is removed effectively irrespective of the temperature, unlike BaP, which is not partitioned easily into the aqueous solution (KOW = 6.13). This is consistent with the soil washing experiments conducted at the bench-scale level. The increase in temperature did not improve the contaminant removal [22].

A similar trend was observed in phthalates. The comparable values of LogWorth for phthalates, as seen in Figure S2, highlighted that all the variables and their cross-effects played an equally significant role in removing them from the fabric. This was primarily because the phthalates in the mix had a stronger interaction with the fabric as compared to the surfactant solution. The effect of increasing temperature on washing efficiency for phthalates was slightly negative, although the results were comparable. The removal of phthalates was consistently low, irrespective of the combination of parameters. This indicated that temperature can only benefit if the surfactants are effective in the first place.

For phenols, the interaction plots are shown in Figure S6. For phenol and 2,4,6-TCP, the change in temperature did not affect their removal and demonstrated consistent washing efficiency. So, the solubilization of the phenol and 2,4,6-TCP by forming salts with the counterion of the surfactants was not affected. For PCP, there was a slight decline in washing efficiency. This was the only significant impact temperature had while removing phenols. Similar to PAHs, the washing efficiency of PCP decreased by increasing the temperature when other parameters were kept constant.

#### 3.2.3. Time

Based on the effect summaries (Figure 4), it was found that the primary limitation of conventional surfactants is their ability to remove phthalates from fabric, even with longer washing durations, due to the superhydrophobic nature of phthalates. The primary reason can be seen in the interaction plots (Figure S5) for the cross-effect of surfactant and time (highlighted in green). CD2 worked better in removing phthalates as compared to CD1 for 15 min of washing but is unable to prevent the redeposition of phthalates on the fabric surface for longer washing durations. The effect of time on CD1 is negative for the removal of DBP, which indicated that DBP started redepositing for longer washing durations. Although higher temperatures and longer washing times together (highlighted in red) showed a positive slope for the removal of phthalates, the removal efficiency values did not increase above 50%. For PAHs, temperature and time had a strong interaction. The third column in Figure S5 is for the time that highlighted the strong interaction of temperature and time for phenanthrene and pyrene (highlighted in red boxes). For BaP, even surfactants strongly interacted with time (box highlighted in green). The effect of CD1 increased with longer washing durations. This is consistent because the hydrophobicity would take time to desorb BaP from the fabric. Overall, the findings suggest that the choice of surfactant, temperature, and time should be carefully considered to effectively remove contaminants from fabric.

### 3.3. Comparative Analysis with Full-Scale Washing

**Figure 8.**
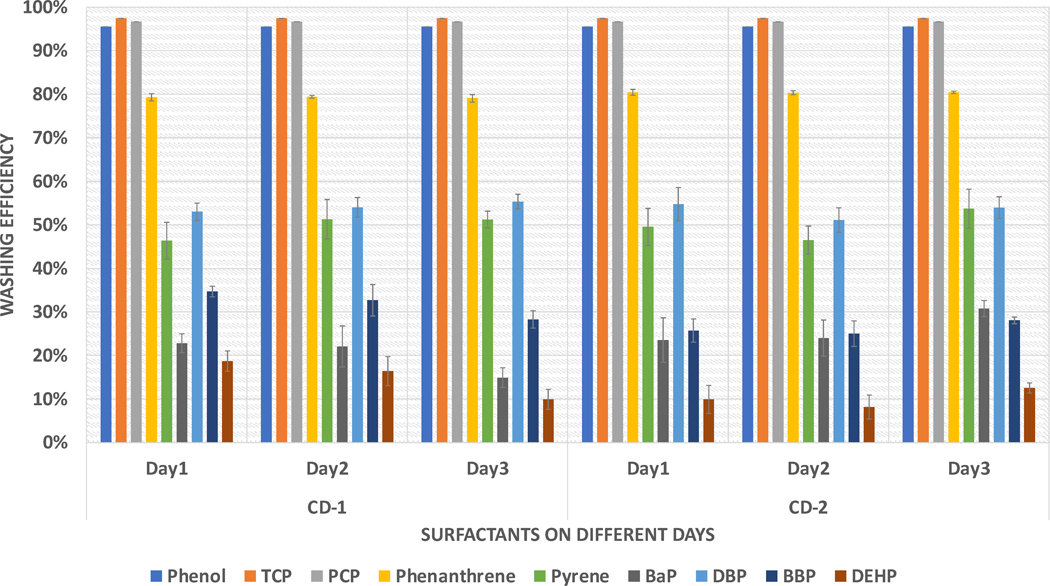
Average washing efficiencies for different combinations (full-scale).

The implementation of full-scale washing was to evaluate the effects of temperature on washing further and understand how it is different from the bench-scale washing experiments. The average washing efficiencies for full-scale DOE are shown in Figure 8. Similar to the bench-scale level, phenols demonstrated higher washing efficiencies than PAHs and phthalates. The washing efficiency of phenol and 2,4,6-TCP was calculated using the LOQ/2 values. The trend of decreasing washing efficiencies as KOW values increased was also consistent at both levels. Similarly, washing efficiencies for BaP and DEHP were consistently low for full-scale washing, which was also observed in bench-scale washing. The values for phenanthrene and DBP in full-scale washing were comparable with those for bench-scale washing, as seen in Figures 3 and 8, respectively. This showed that common trends in chemical groups for full-scale washing can be correctly predicted using bench-scale washing. The average washing efficiencies for all the compounds were higher in full-scale washing when compared with bench-scale. This indicated that the mechanical agitation in the full-scale washer-extractor may have contributed to the removal of contaminants.

**Figure 9.**
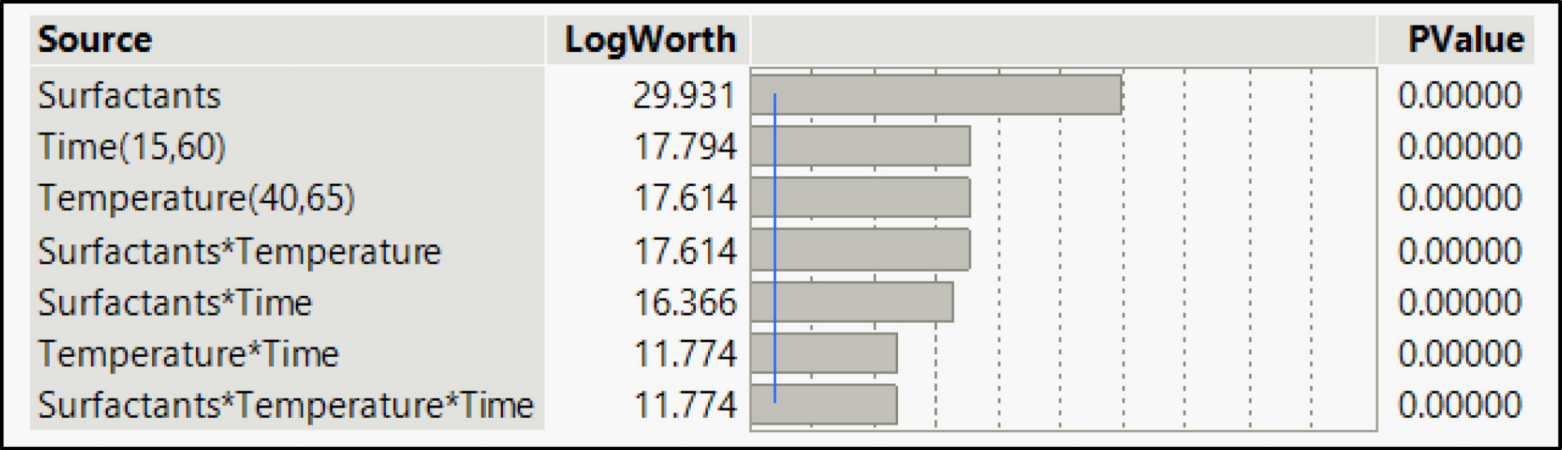
Effect summary for entire mix (full-scale).

Figure 9 shows the effect summary of various washing parameters on the cleaning efficiency for all targeted contaminants. The LogWorth value of surfactant had the highest impact, followed by the cross-effect of surfactant with temperature and the effect of temperature alone. These results were consistent with the bench-scale washing experiments. The effect of time on washing efficiency for full-scale washing increased, which was different from the negative impact observed in the bench-scale experiments. The LogWorth value of parameters in decreasing order of importance was surfactants > temperature > time for PAHs and phthalates. The effect summary indicated that the parameters affected the washing efficiency on full-scale washing differently than on bench-scale washing, and their individual and cross-effects are discussed separately.

#### 3.3.1. Surfactants

**Figure 10.**
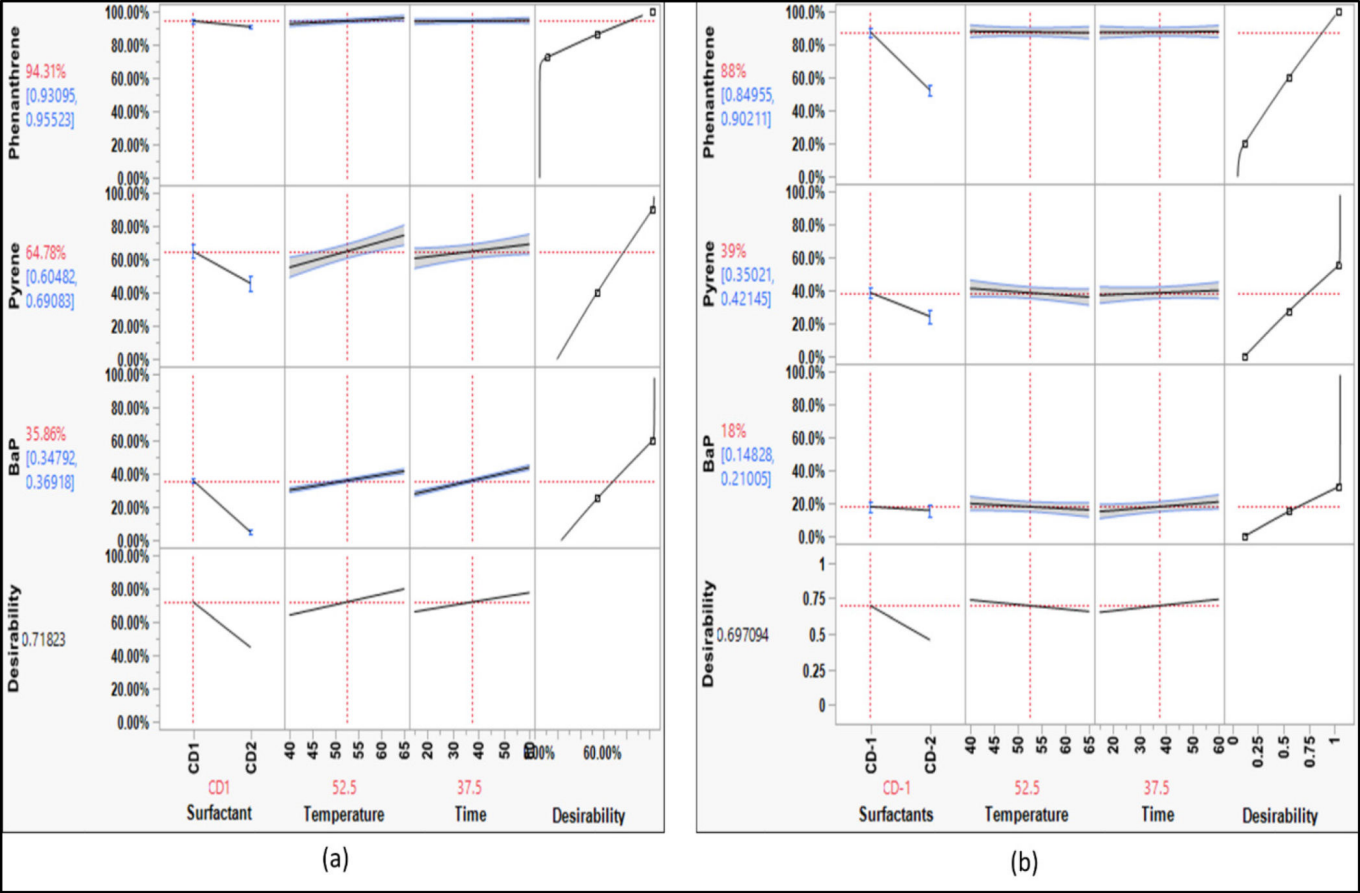
Prediction profile for PAHs: (**a**) full-scale and (**b**) bench-scale.

Figure 10 shows the comparative analysis of prediction profiles for PAHs on both bench and full scales. The surfactant CD1 effectively removed PAHs, which is consistent with the results of the bench-scale washing. However, there was a difference in the contaminant-to-surfactant volume ratio between the two scales. In the bench-scale experiments, each contaminant was present in 60,000 ng, and 45 μL of surfactant solution was injected into a single flask, resulting in a ratio of 1333.34 ng of contaminant per μL of surfactant. In the full-scale experiments, this ratio decreased to 2.5 ng of contaminant per μL of surfactant. The higher concentration of surfactants in the full-scale experiments increased the micelle concentration, producing more hydrophobic cavities that adsorbed more contaminants from the fabric. This led to better removal of contaminants. In addition, the increase in G-force increased mechanical agitation, which further helped in the removal of contaminants.

**Figure 11.**
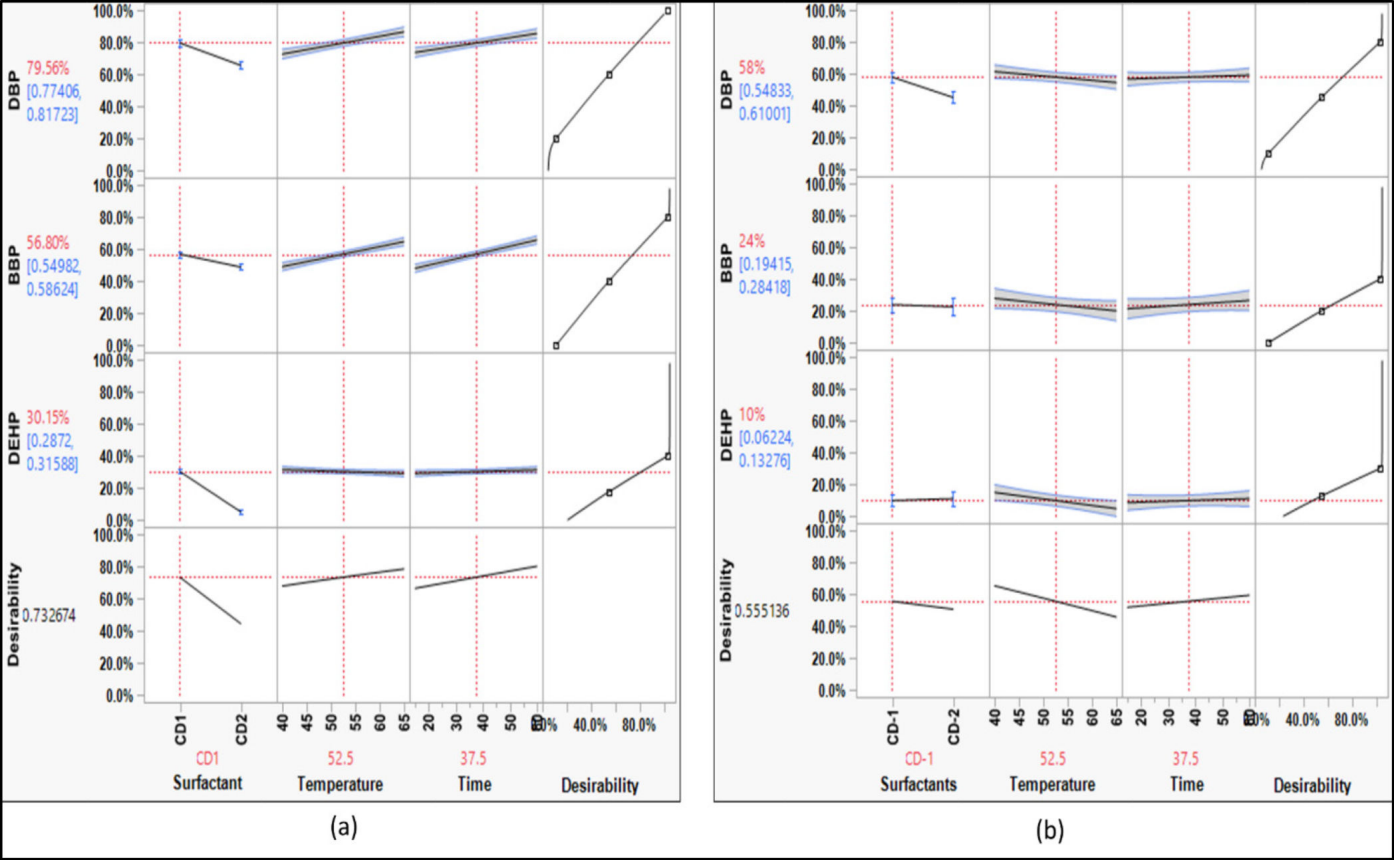
Prediction profile for phthalates: (**a**) full-scale and (**b**) bench-scale.

For phthalates (Figure 11), the washing efficiency for CD1 was higher, which was similar to PAHs. Thus, the non-polar nature of the compounds needs to be considered. The results showed that for non-polar compounds such as phthalates, CD1 was more effective in removing contaminants than CD2. DEHP, a hydrophobic phthalate, showed little sensitivity to changes in surfactant concentration in the full-scale experiments. The mixed ionic surfactant system in CD2 was not effective in removing phthalates. Therefore, for non-polar compounds, CD1 was more effective in removing phthalates, which is consistent with the bench-scale washing experiments.

**Figure 12.**
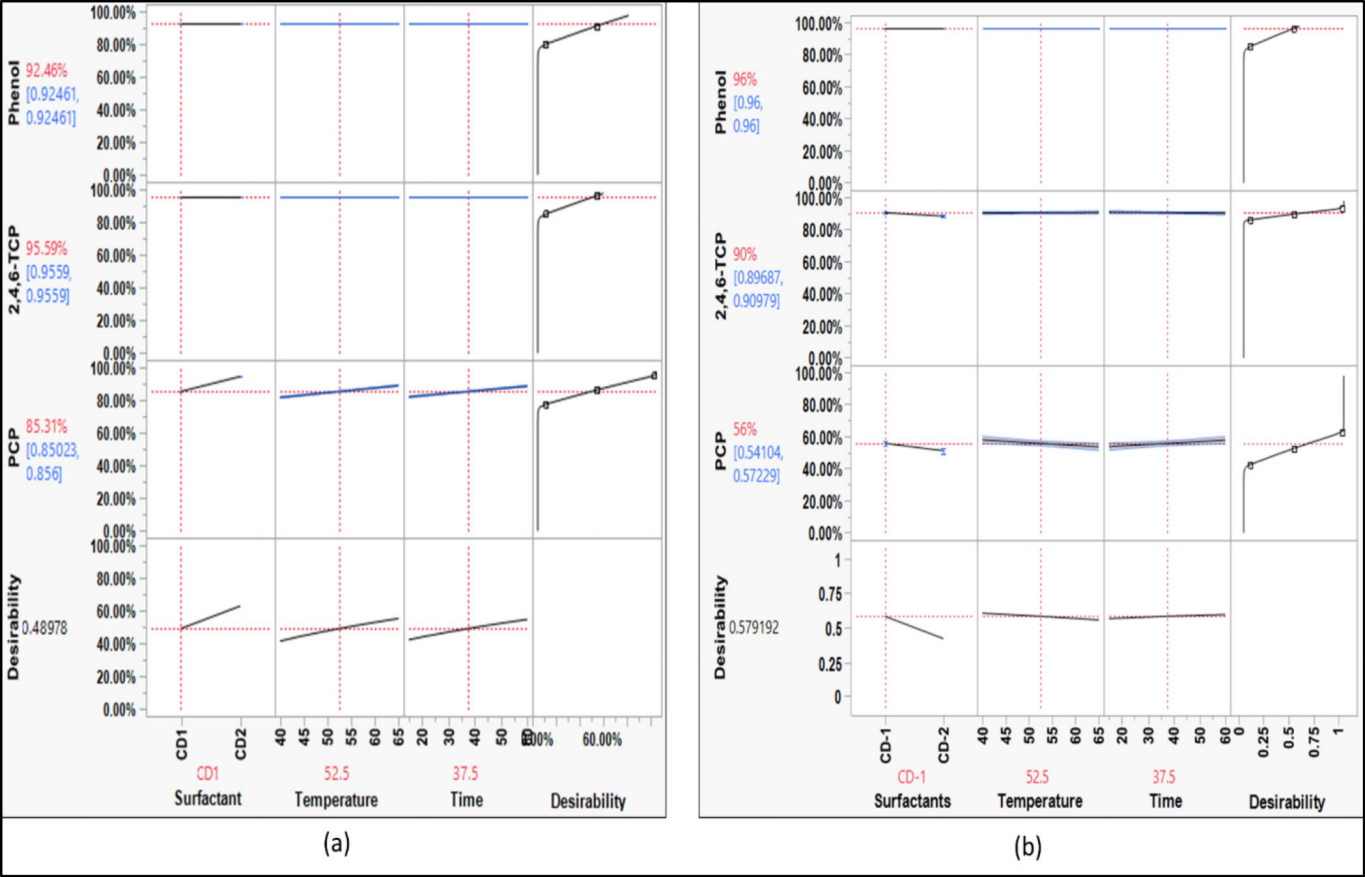
Prediction profile for phenols: (**a**) full-scale and (**b**) bench-scale.

Although both surfactants were effective in removing phenol and 2,4,6-TCP, PCP responded positively to CD2 used for a longer washing duration at a higher temperature, although the results were comparable (Figure 12). This may be due to the higher concentration of anionic surfactants that enabled solubilizing the PCP by forming salts with the counterion.

#### 3.3.2. Temperature

The impact of temperature on the effectiveness of washing was found to be positive for all three classes of contaminants, which was different from the bench-scale washing experiments. The high concentration of surfactants available during the full-scale washing process played a key role in the efficient removal of contaminants. Moreover, the significant temperature variations experienced during the washing cycle may have further contributed to the removal of contaminants. The higher temperature of the surfactant solution during full-scale washing may have also approached the cloud point, which is the temperature at which non-ionic surfactants have maximum surface activity. This may have helped to remove the contaminants more effectively.

The interaction plots for PAHs (Figure S7) showed that increasing temperature had a positive effect on the efficiency of both surfactants (CD1 and CD2). Similarly, the efficiency of surfactants for phthalates (Figure S8), especially DBP and BBP, gradually increased with an increase in temperature. However, DEHP was found to be unresponsive to higher temperatures both in bench-scale and full-scale washing, as it is not easily partitioned into water.

This indicates that the interaction between surfactants and contaminants is crucial for hydrophobic compounds, and other parameters have a secondary or complementary effect on their removal. The washing efficiency was consistent for the surfactant temperature curve for DEHP in the interaction plots (Figure S8).

The effect of temperature on phenols was not significant, primarily because the phenols had a strong interaction with surfactants and were removed efficiently. The response curve was primarily driven by PCP, which indicated a slight decline in its removal with an increase in temperature, though the results were still comparable. For PCP, there was a weak interaction of CD1 with temperature and time (green highlighted boxes) in Figure S9.

#### 3.3.3. Time

For full-scale washing, the effect of time showed positive results for most of the targeted contaminants. This was likely due to the higher availability of surfactant molecules present in the washing solution, which facilitated the desorption of the contaminants from the fabric. Specifically, pyrene and BaP showed significant improvements in washing efficiency with longer washing times, as indicated in Figure 10. The interaction plots for PAHs demonstrated that the washing efficiency of the surfactants improved drastically for pyrene, while for BaP it improved gradually, suggesting that these compounds take longer to be removed from the fabric.

Similar results were observed for the removal of phthalates, where longer washing durations resulted in improved removal of DBP and BBP. Figure S8 illustrates that the washing efficiency of surfactants for phthalates increased gradually with longer washing durations. However, for DEHP, the surfactant curve remained consistent, indicating that the current surfactants can remove only a limited amount of DEHP regardless of the washing duration.

The effect of time on the removal of phenols was consistent with bench-scale washing, where longer washing durations resulted in a slight increase in the washing efficiency for CD1 to remove pentachlorophenol. This suggests that phenols are washed out at shorter durations when the effect of surfactant is strong.

It is important to note that from the bench-scale washing experiments, the possibility of redeposition of the contaminants was observed. It is possible that the contaminants might be redeposited on other parts of the jackets other than the swatches, which could affect the overall calculations of washing efficiency.

## 4. Conclusions

In this study, we aimed to evaluate the effectiveness of various washing parameters in removing targeted fireground contaminants from firefighting PPE using a bench-scale approach. We also assessed the comparability between bench-scale testing and full-scale laundering practices regarding their ability to evaluate the cleaning efficacy of firefighting PPE.

Firstly, we found that the bench-scale washing method was consistent and could be used to evaluate the cleaning efficacy of firefighting PPE. For bench-scale washing experiments, the results showed that different parameters affected the washing efficiency of other chemicals, and all parameters and their cross-effects impacted cleaning. This highlighted that decontaminating outershell material is a problem with no single solution. It also indicates that when evaluating the cleaning efficacy of an independent service provider, the mean (arithmetic average) of cleaning efficacy for individual chemical groups should be considered rather than an overall average.

The surfactant’s effect was significant on cleaning efficiency, with the non-ionic detergent CD1 demonstrating better results for PAHs and phthalates than CD2. An inverse relationship was observed between KOW values and washing efficiency, with low removal of BaP and DEHP. Our study also showed a very limited scope for removing complex PAHs and phthalates using conventional surfactants, and the development of new surfactants will allow for more testing possibilities.

Secondly, we found that the effectiveness of laundering practices in removing contaminants from firefighting PPE is highly dependent on the specific contaminants and the parameters used. While the effect of temperature individually on the cleaning efficiency was not significant for the targeted contaminants, the interaction plots indicated that a longer washing duration and a higher temperature could improve the washing efficiency for some chemicals. Although the effect of higher temperatures and longer washing durations on the durability of the outershell material needs to be studied, the effect of time worked in both directions, with complex PAHs such as BaP being removed when the surfactant solution was in contact with the fabric for longer washing durations, while phthalates such as DBP and BBP were redeposited on the fabric.

Thirdly, we found that the full-scale washing experiments showed that the temperature variation in the washer-extractor was high and that the volume of surfactant and water used contributed to the removal of contaminants. All the trends shown in the bench-scale experiments were seen in the full-scale washing experiments. On both levels of experiments, it was shown that as the hydrophobicity of the compound increases, the removal of contaminants from the fabric becomes more difficult. The overall washing efficiency of all the contaminants increased for all the experiments in full-scale washing. However, the conventional wash (wash according to the NFPA 1851 guidelines) did not effectively remove contaminants such as BaP and DEHP, while simpler PAHs such as phenanthrene and phthalates such as DBP were effectively removed. This was significantly highlighted for phthalates, where there was low contaminant removal, irrespective of the parameters.

Our study has some limitations, including the absence of G-force in the bench-scale washing method and the potential to migrate contaminants from swatches to other parts of the garment in the full-scale washing method. Future research should consider these limitations and investigate the redeposition of contaminants as well as the development of specialized cleaning methods for removing residual contamination. Additionally, the following research needs to be conducted on the inner layers, such as the moisture barrier and thermal liners, to assess the effect of different parameters on cleaning efficacy in these layers.

In conclusion, our study provides valuable insights into the effectiveness of laundering practices for removing targeted fireground contaminants from firefighting PPE. The study highlights the complexity of contaminant removal and the need for specific cleaning methods for different contaminants. The study also demonstrates the usefulness of bench-scale testing in evaluating the cleaning efficacy of different surfactants used to clean firefighting PPE and provides guidance on optimizing laundering practices to reduce the risks of occupational chemical exposure for firefighters, ultimately improving their health and safety.

## Supplementary Material

Supplementary Figures

## Figures and Tables

**Table 1.** Targeted contaminants and their relevant properties.

Compound	Boiling Point (°C)	Volatile/Semi-Volatile	KOW ^A^	LOD (ng/μL)	LOQ (ng/μL)	RSQ
Phenol	182	Volatile	1.46	0.29	0.90	0.9988
2,4,6-Trichlorophenol (2,4,6-TCP)	246	Volatile	3.69	0.17	0.52	0.9973
Pentachlorophenol (PCP)	310	Semi-volatile	5.12	0.22	0.67	0.9927
Di-butyl phthalate (DBP)	340	Semi-volatile	4.50	0.09	0.26	0.9998
Benzyl butyl phthalate (BBP)	370	Semi-volatile	4.73	0.10	0.30	0.9994
Di-ethylhexyl phthalate (DEHP)	384	Semi-volatile	7.60	0.13	0.38	0.9997
Phenanthrene	340	Semi-volatile	4.46	0.22	0.67	0.9992
Pyrene	404	Semi-volatile	4.88	0.07	0.21	0.9997
Benzo[a] pyrene (BaP)	495	Semi-volatile	6.13	0.06	0.18	0.9995

A = values taken from PubChem^®^.

**Table 2.** Experimental design for testing consist

Day 1		Day 2		Day 3	
CD-l	CD-2	CD-l	CD-2	CD-l	CD-2
Sample-1	Sample-l	Sample-4	Sample-4	Sample-7	Sample-7
Sample-2	Sample-2	Sample-5	Sample-5	Sample-8	Sample-8
Sample-3	Sample-3	Sample-6	Sample-6	Sample-9	Sample-9

**Table 3.** Parameters for the design of experiments.

Surfactants	Temperature (°C)	Time (min)
CD1	40	15
CD2	65	60

**Table 4.** Ingredients for CD1 and CD2.

CD1	CD2
D-Limonene	Non-ionic surfactant, Alcohol ethoxylate
Non-ionic surfactant: 4-Nonylphenyl-polyethylene glycol	Anionic surfactant: Alkyl ethoxy sulfate and alkyl sulfate, linear alkylbenzene sulfonate
Mackamide C	Amine oxide
Glycol ether	Hydrogen peroxide
	Percarbonate

## Data Availability

All the data supporting the findings of the study are available within the article.
