# Supplementary material for: Bench-Scale and Full-Scale Level Evaluation of the Effect of Parameters on Cleaning Efficacy of the Firefighters’ PPE
Source: Textiles (Basel). Author manuscript; Available in PMC 2023 Oct 2. (PMC10543714; doi:10.3390/textiles3020014)
Supplement: Supplementary Figures [file NIHMS1925738-supplement-Supplementary_Figures.pdf]

Source	LogWorth	PValue
Surfactants	11.923	0.00000
Temperature(40,65)	7.705	0.00000
Surfactants*Temperature	7.419	0.00000
Temperature*Time	4.276	0.00005
Time(15,60)	2.949	0.00113
Surfactants*Time	2.654	0.00222
Surfactants*Temperature*Time	2.555	0.00279

Figure S1: Effect summary for PAHs (bench-scale)

Source	LogWorth	PValue
Temperature(40,65)	5.694	0.00000
Temperature*Time	4.897	0.00001
Surfactants	4.190	0.00006
Surfactants*Temperature	3.490	0.00032
Surfactants*Temperature*Time	2.626	0.00237
Surfactants*Time	0.722	0.18964
Time(15,60)	0.348	0.44924

Figure S2: Effect summary of phthalates (bench-scale)

Source	LogWorth	PValue
Temperature(40,65)	3.875	0.00013
Surfactants	3.331	0.00047
Temperature*Time	1.546	0.02846
Time(15,60)	1.336	0.04615
Surfactants*Time	1.260	0.05499
Surfactants*Temperature*Time	0.868	0.13547
Surfactants*Temperature	0.657	0.22051

Figure S3: Effect summary for phenols (bench-scale)

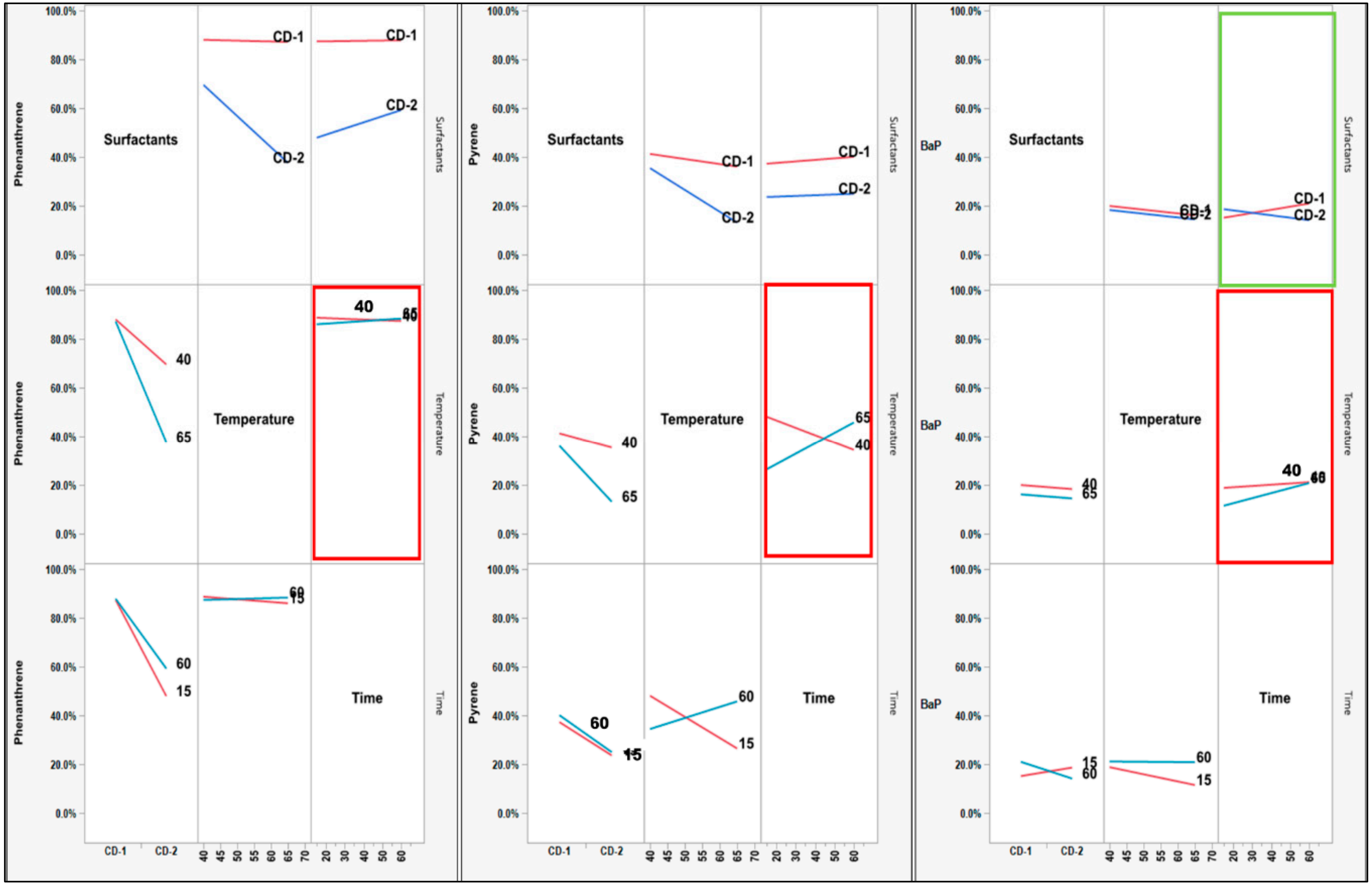

Figure S4: Interaction plots for PAHs (Bench-scale)

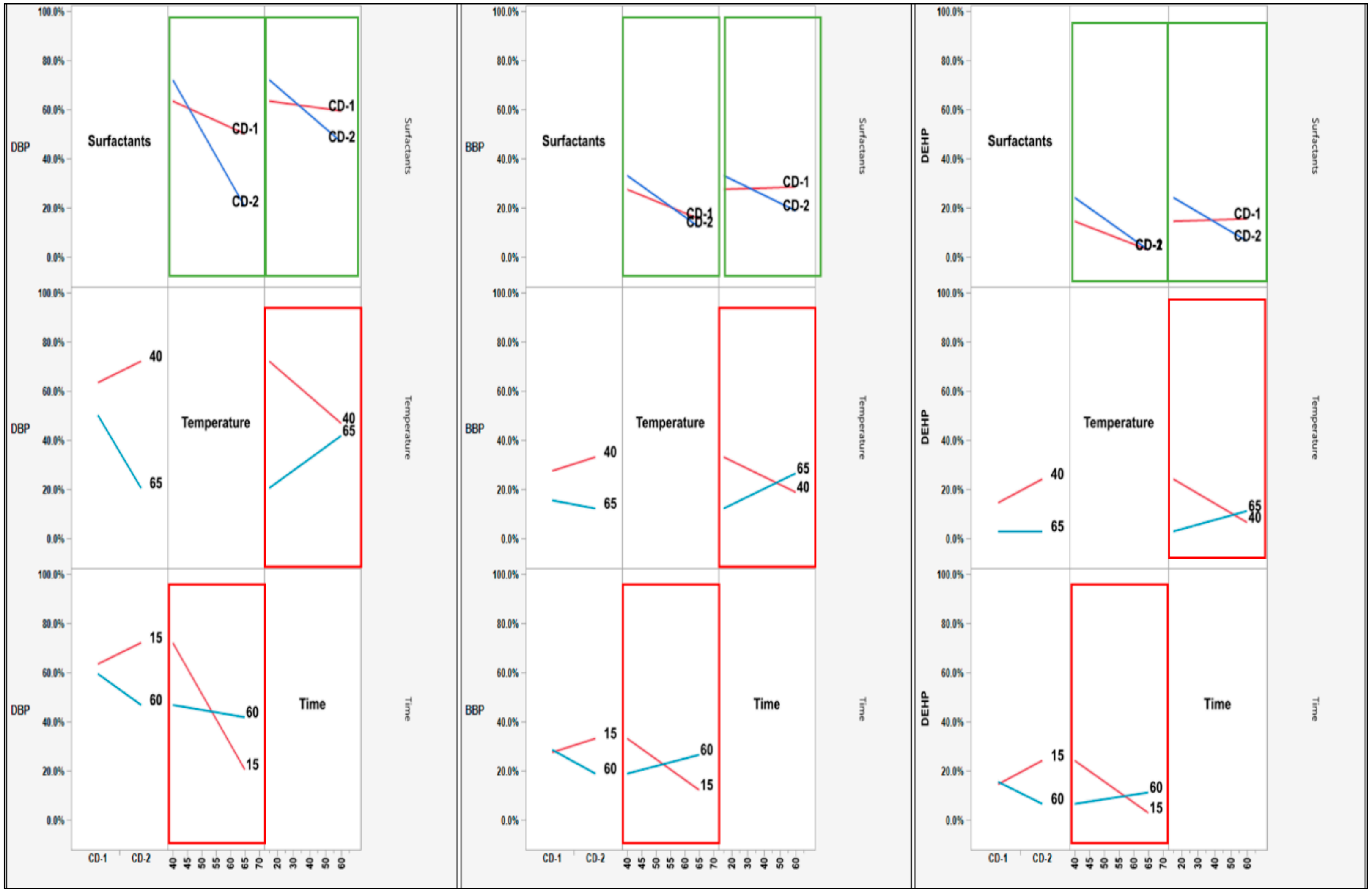

Figure S5: Interaction plots for phthalates (Bench-scale)

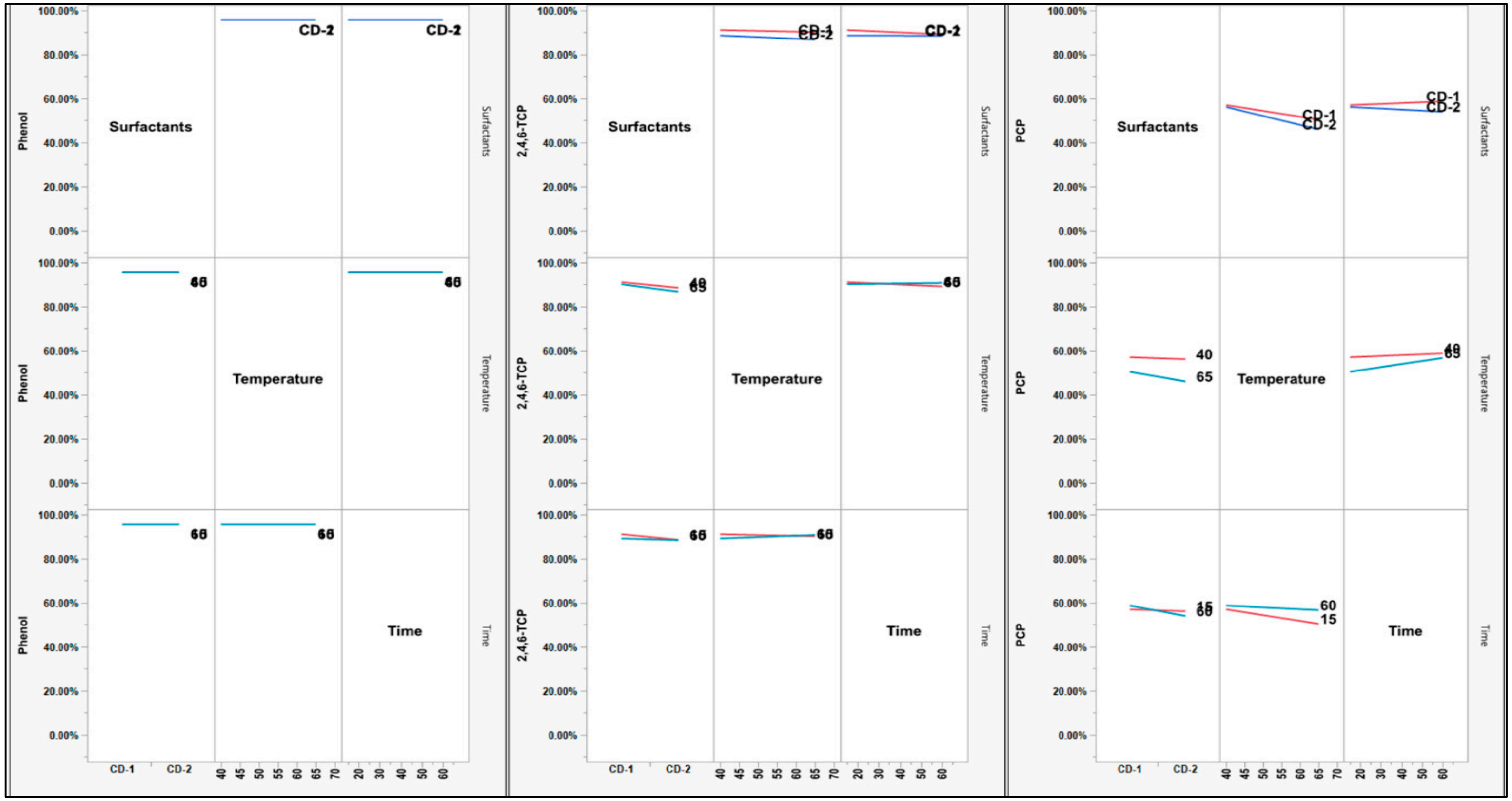

Figure S6:Interaction plots for phenols (Bench-scale)

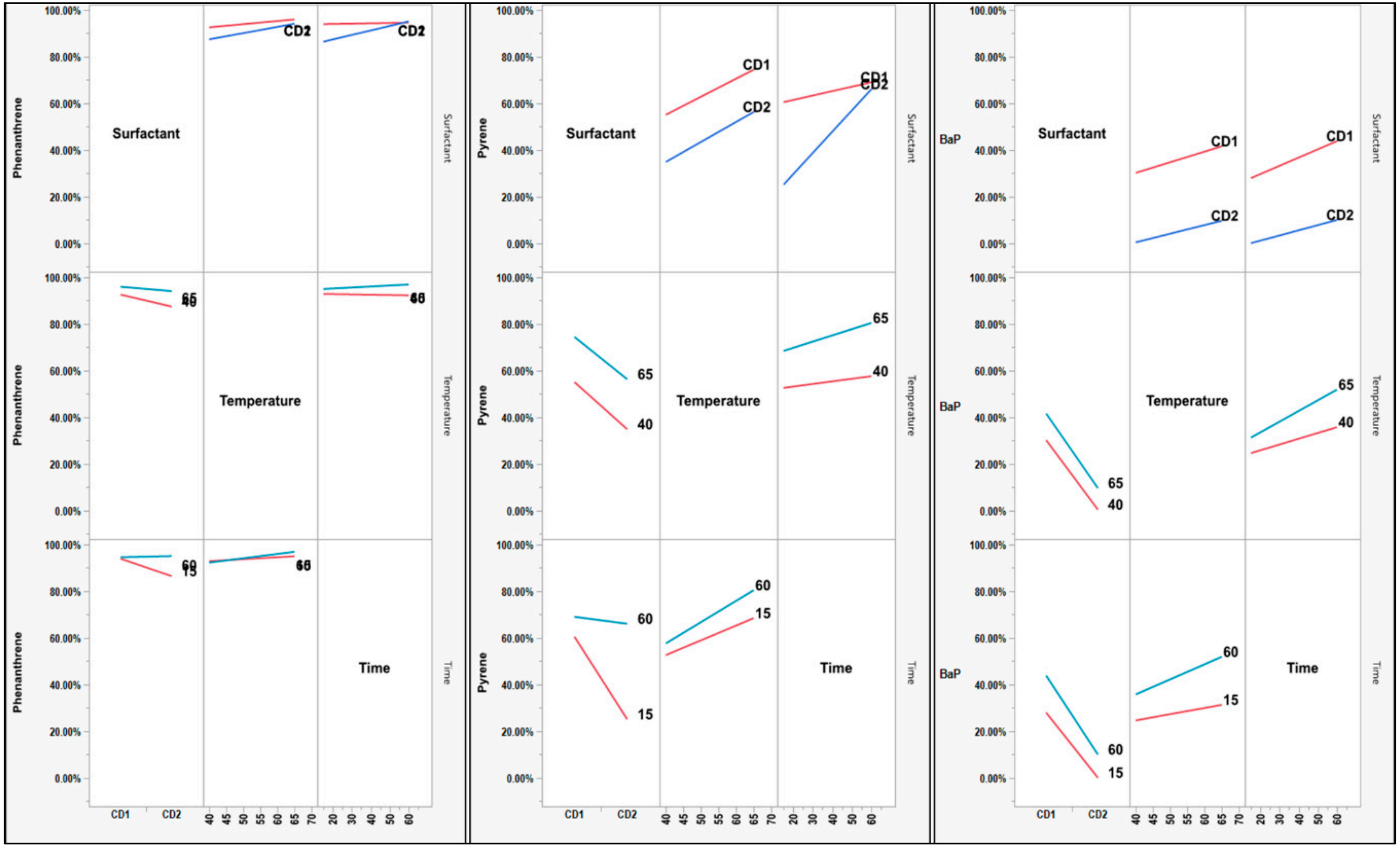

Figure S7: Interaction plots for PAHs (Full-scale)

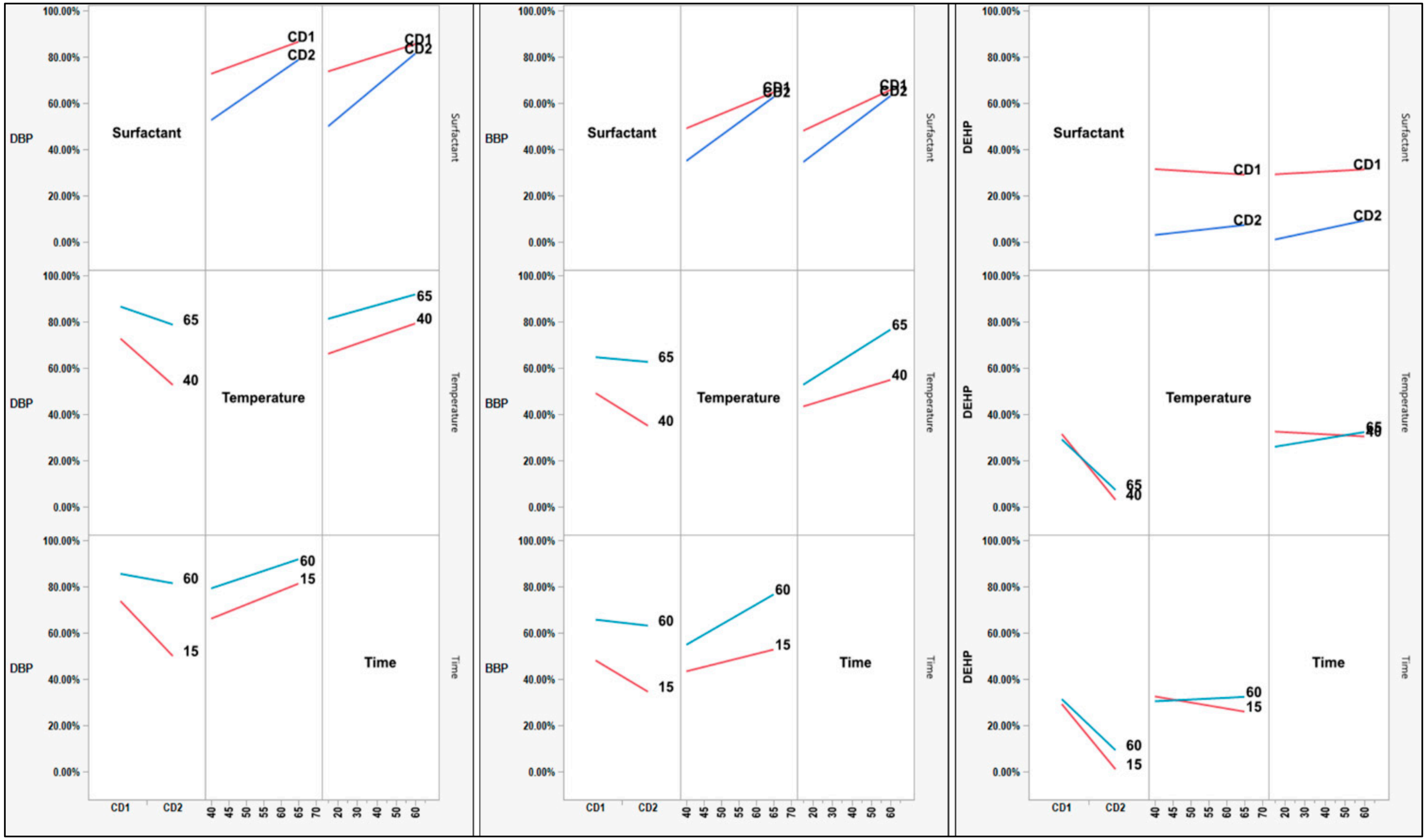

Figure S8: Interaction plots for phthalates (Full-scale)

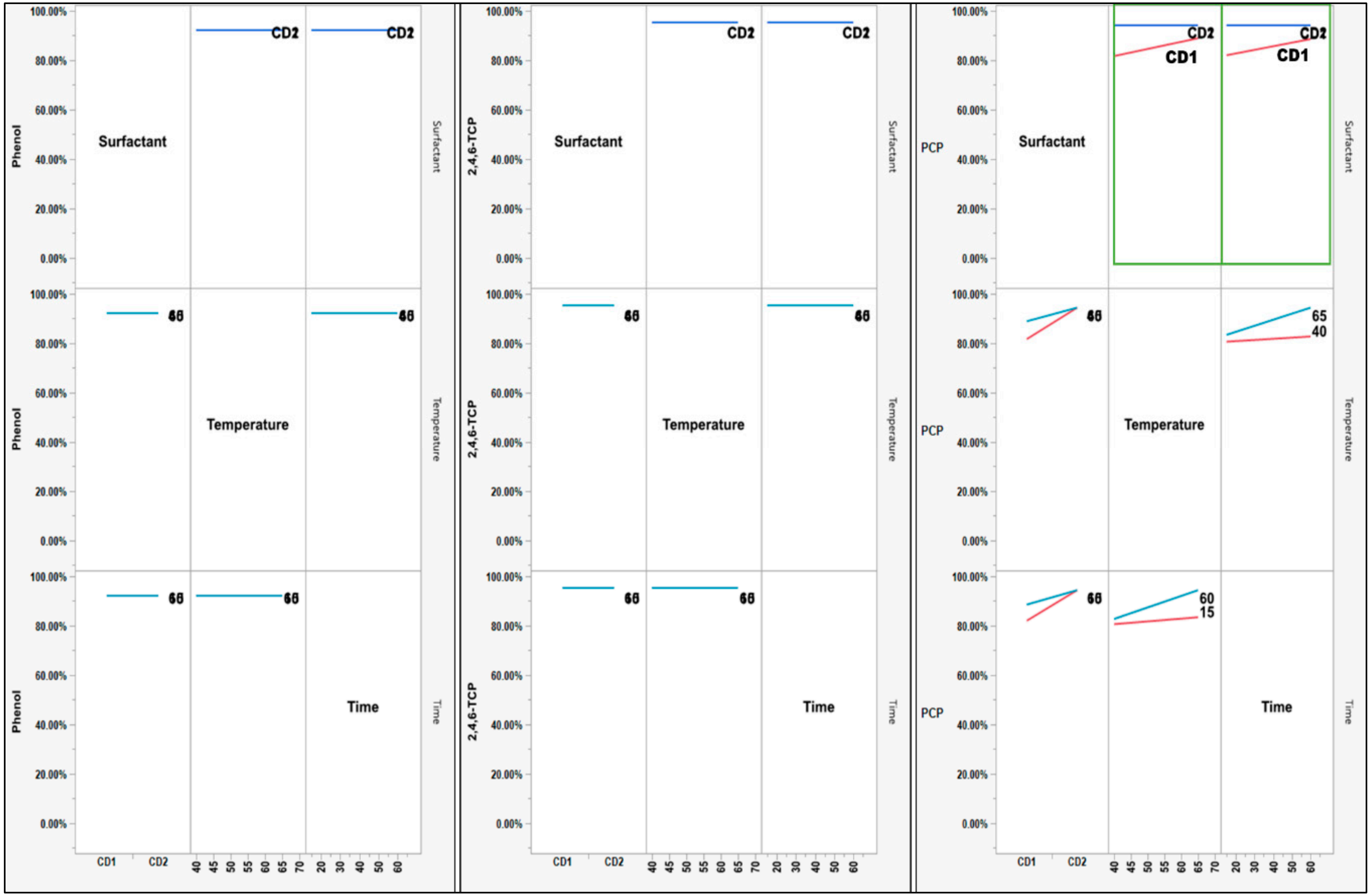

Figure S9: Interaction plots for phenols (Full-scale)
